# Neutrophil to lymphocyte ratio varies in magnitude and biomarker utility based on patient demographics

**DOI:** 10.1172/JCI198948

**Published:** 2025-11-11

**Authors:** William Ang, Travis D. Kerr, Ananya Kodiboyena, Cristina Valero, Joris L. Vos, Vladimir Makarov, Alex A. Adjei, Luc G.T. Morris, Stephanie L. Schmit, Natalie L. Silver, Sujata Patil, Daniel J. McGrail

**Affiliations:** 1 Center for Immunotherapy and Precision Immuno-Oncology, Cleveland Clinic, Cleveland, Ohio, USA.; 2 Cleveland Clinic Lerner College of Medicine, Case Western Reserve University, Cleveland, Ohio, USA.; 3 Quantitative Health Sciences, Cleveland Clinic, Cleveland, Ohio, USA.; 4 Head and Neck Service, Department of Surgery, Memorial Sloan Kettering Cancer Center, New York, New York, USA.; 5 Taussig Cancer Institute,; 6 Genomic Medicine Institute, and; 7 Head and Neck Institute, Cleveland Clinic, Cleveland, Ohio, USA.

**Keywords:** Oncology, Public Health, Biomarkers, Cancer immunotherapy, Population health research

## Abstract

Ang et al. show that a widely studied biomarker for immunotherapy varies based on patient demographics, and that it may only predict outcomes in certain populations.

**To the Editor:** The neutrophil to lymphocyte ratio (NLR) in the peripheral blood is viewed as a marker of inflammation and has been associated with poor outcomes in a variety of diseases (1). An increasing emphasis has been placed on the potential for the NLR to predict outcomes following treatment with immune checkpoint inhibition (ICI) immunotherapies (2). Despite evidence that patient demographics may impact blood test reference values (3) and the utility of ICI biomarkers such as tumor mutation burden (4), there has been minimal study into how the NLR varies by patient demographics and whether the NLR predicts ICI outcomes across different population subsets. This paper presents evidence that the NLR varies by race/ethnicity and sex, and that the association of the NLR with ICI outcomes is restricted to non-Hispanic White (NHW) individuals.

Associations between patient demographic features and the NLR were analyzed in the nationally representative National Health and Nutrition Examination Survey (NHANES, Supplemental Table 1; supplemental material available online with this article; https://doi.org/10.1172/JCI198948DS1). We found that the NLR was elevated in men and increased with age, BMI, and inflammation (see Supplemental Methods), and decreased in all race/ethnicity groups compared with NHW (Figure 1A). Upon multivariable analysis, all covariates retained significance except BMI (Figure 1B).

Based on our observation that the NLR was significantly altered by sex and race/ethnicity, we hypothesized that different threshold values for high NLR based on patient demographics may be required to optimally stratify ICI outcomes. Using data from Memorial Sloan Kettering Cancer Center (MSKCC) (5), we divided patients into high and low NLR by splitting at the upper tertile of NLR values. As expected, this resulted in a strongly significantly worse progression-free survival (PFS) in NHW patients with a high NLR (Figure 1C). Repeating this analysis with patients from other racial/ethnic groups, we observed no association with PFS, including for non-Hispanic Black (NHB) (Figure 1C), Asian (Supplemental Figure 1A), and Hispanic patients (Supplemental Figure 1B).

To determine whether alternative thresholds could significantly stratify patients across populations, we next evaluated the association of NLR with ICI PFS using a range of thresholds. We found that NLR was significantly associated with PFS following ICI across all analyzed thresholds for NHW patients, but that neither NHB nor Asian cohorts achieved significance at any NLR threshold value assessed when either analyzing all available patients (Figure 1D) or down-sampling all cohorts to equal sample size (Supplemental Figure 1C), suggesting ICI outcomes may only be associated with the NLR in patients with NHW.

To validate our observation that the NLR was only associated with ICI outcomes in NHW individuals in individual cancer types, we compiled a new cohort of 5,128 patients treated with ICI from the Cleveland Clinic Foundation (CCF). We first analyzed patients with lung cancer treated with ICIs, which accounted for 50% of the initial MSKCC cohort. We found a comparable ability to stratify PFS in NHW patients, but no association in NHB, Asian, or Hispanic patients (Figure 1E and Supplemental Figure 2A). Based on the NHW hazard ratio, the NHB cohort had 83% power to detect a significant association between NLR and PFS on ICIs, suggesting lack of significance was not due to sample size. The NLR was associated with ICI PFS in NHW patients with breast cancer, but again no significant association with PFS was found in NHB patients at 89% power (Figure 1F). Similar trends were observed with kidney/renal cancer (Figure 1G and Supplemental Figure 2B), liver cancer (Figure 1H and Supplemental Figure 2C), cervical cancer (Figure 1I), and head and neck cancer (Figure 1J). Pooling all cancer types, we still failed to detect an association between NLR and ICI outcomes in data from NHB patients at 99% power (Supplemental Figure 2D). Likewise, extending this model to include all patients with an interaction effect found a significant interaction effect between NLR and NHB race/ethnicity (Supplemental Figure 2E). Upon analyzing patients treated with non-ICI regimens, we did not identify differences by race/ethnicity, suggesting this phenomenon is specific to ICI (Supplemental Figure 2, F and G).

To test whether sex-based differences in baseline NLR may impact the optimal threshold to predict ICI outcomes within NHW patients, we first determined optimal threshold values for both male and female NHW patients with lung cancer from the MSKCC cohort, finding 6.3 and 9.5, respectively. We visualized ICI outcomes stratified into low NLR, NLR between the female and male threshold, and NLR higher than the male threshold. ICI outcomes were worse for female patients with NLR values above either threshold, but for male patients worse prognosis was only observed above the male threshold (Supplemental Figure 3A). This result was confirmed in CCF cohorts of patients with lung cancer, kidney cancer, liver cancer, and head and neck cancer (Supplemental Figure 3, B–E).

In conclusion, our study demonstrates that the NLR varies based on sex and race/ethnicity, the association of NLR with ICI outcomes was restricted to NHW individuals, and that within NHW individuals the optimal threshold varied by sex (see Supplemental Discussion). Together, these observations highlight the importance of considering patient demographics when implementing clinical biomarkers.

## Funding support

This work is the result of NIH funding, in whole or in part, and is subject to the NIH Public Access Policy. Through acceptance of this federal funding, the NIH has been given a right to make the work publicly available in PubMed Central.

- National Cancer Institute grant R37CA295609 (to DJM).
- VeloSano 9 (to DJM).
- American Cancer Society grants RSG-24-1322538 (to NLS) and R01CA248931 (to SLS).

## Supplementary Material

Supplemental data

Supporting data values

## Figures and Tables

**Figure 1:**
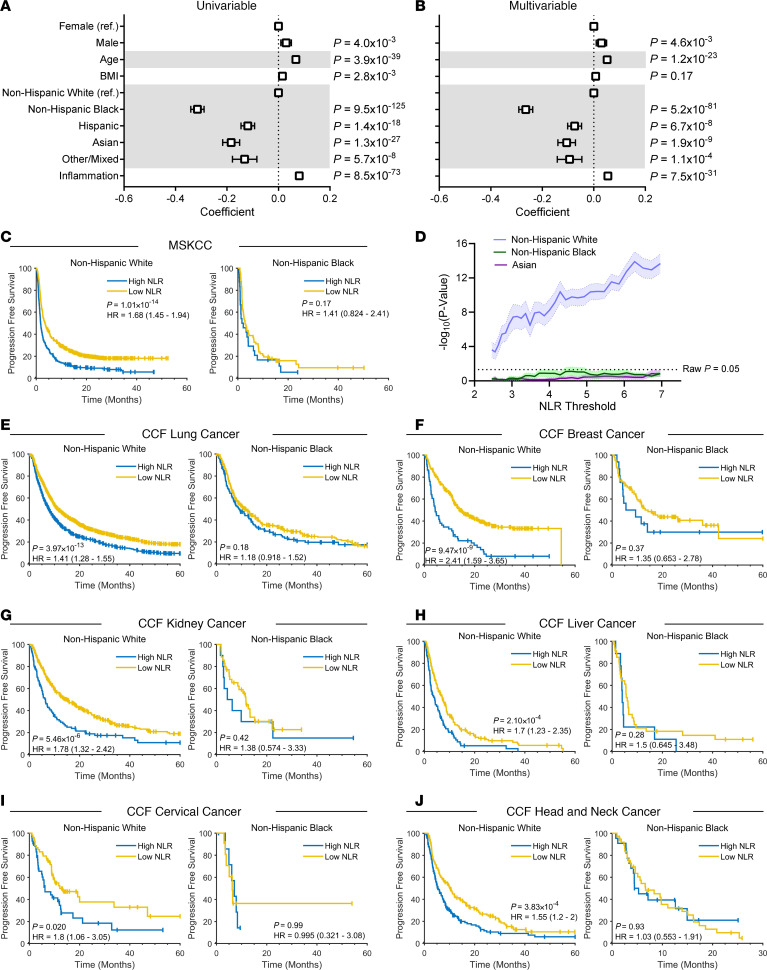
NLR varies in magnitude and prognostic relevance by patient race/ethnicity and sex. (**A** and **B**) Univariable (**A**) and multivariable (**B**) regression model between indicated variables and log_2_(NLR). *N* = 8,095. Box-and-whisker plots show the regression coefficient with 95% confidence interval. (**C**) Kaplan-Meier curves following ICI treatment for NHW (*N* = 1,135) and NHB patients (*N* = 91) from MSKCC. (**D**) Significance determined by log-rank test as function of NLR threshold values in patients from MSKCC. Shaded region indicates standard deviation from cross-validation. NHW *N* = 1,135, NHB *N* = 91, Asian *N* = 96. (**E**–**J**) Kaplan-Meier curves following ICI treatments in patients from CCF with (**E**) lung cancer, NHW *N* = 2550, NHB *N* = 49; (**F**) breast cancer, NHW *N* = 352, NHB *N* = 102; (**G**) kidney cancer, NHW *N* = 508, NHB *N* = 46, (**H**) liver cancer, NHW *N* = 348, NHB *N* = 57; (**I**) cervical cancer, NHW *N* = 109, NHB *N* = 21; and (**J**) head and neck cancers, NHW *N* = 408, NHB *N* = 71. For all Kaplan-Meier curves, log-rank *P* values are shown with values split at upper tertile of cohort. HR, hazard ratio.

## Version 1 11/11/2025

In-Press Preview

## Version 2 01/02/2026

Electronic publication
