# Supplementary material for: Neutrophil to lymphocyte ratio varies in magnitude and biomarker utility based on patient demographics
Source: J Clin Invest. 2025 Nov 11;136(1):e198948. doi: 10.1172/JCI198948 (PMC12721878; doi:10.1172/JCI198948)
Supplement: Supplemental data [file jci-136-198948-s044.pdf]

Supplemental material for:

Neutrophil to lymphocyte ratio varies in magnitude and biomarker utility based on patient demographics

William Ang[1,2], Travis D. Kerr[1], Ananya Kodiboyena[3], Cristina Valero[4], Joris L. Vos[4], Vladimir Makarov[1], Alex A. Adjei[5], Luc G.T. Morris[4], Stephanie L. Schmit[2,6], Natalie L. Silver[1,7], Sujata Patil[3,5], Daniel J. McGrail[1,2*]

[1]Center for Immunotherapy and Precision Immuno-Oncology, Cleveland Clinic, Cleveland, OH, USA
[2]Cleveland Clinic Lerner College of Medicine, Case Western Reserve University, Cleveland, OH, USA
[3]Quantitative Health Sciences, Cleveland Clinic, Cleveland, OH, USA
[4]Head and Neck Service, Department of Surgery, Memorial Sloan Kettering Cancer Center, New York, NY, USA.
[5]Taussig Cancer Institute; [6]Genomic Medicine Institute; [7]Head and Neck Institute, Cleveland Clinic, Cleveland, OH, USA.

*Correspondence to Daniel J. McGrail (mcgraid@ccf.org)

Figures S1-S3
Table S1
Supplemental Discussion
Materials and Methods
Author Contributions
Supplemental References

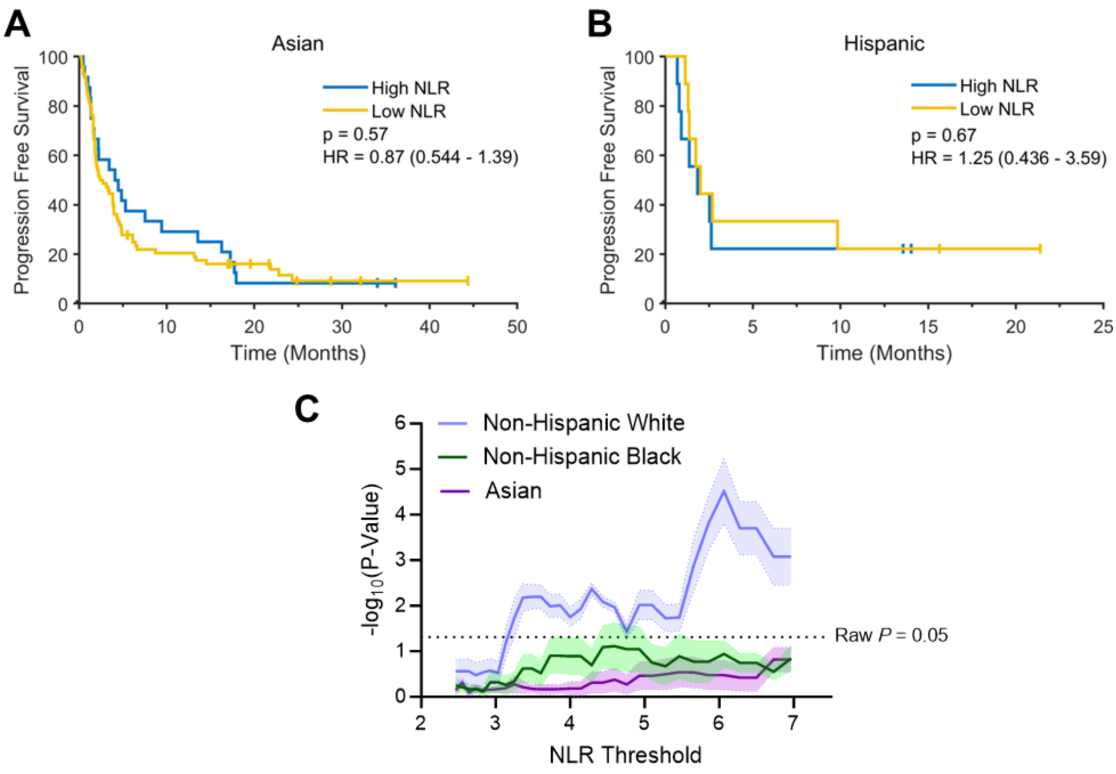

Figure S1. Outcomes following immune checkpoint inhibition in MSKCC cohort stratified by race/ethnicity.

(A-B) Kaplan-Meier curves following treatment with immune checkpoint inhibition for (A) Asian patients (N = 96), and (B) Hispanic patients (N = 18). Log-rank P-values. Values split at upper tertile of cohort. HR, Hazard Ratio.

(C) Significance determined by log-rank test as function of NLR threshold values per Figure 1D, but down-sampling all cohorts to equal sample size to evaluate if significance observed in non-Hispanic white patients is due to higher overall N when using all samples for analysis. Shaded region indicates standard deviation from cross-validation. N = 91 for each group.

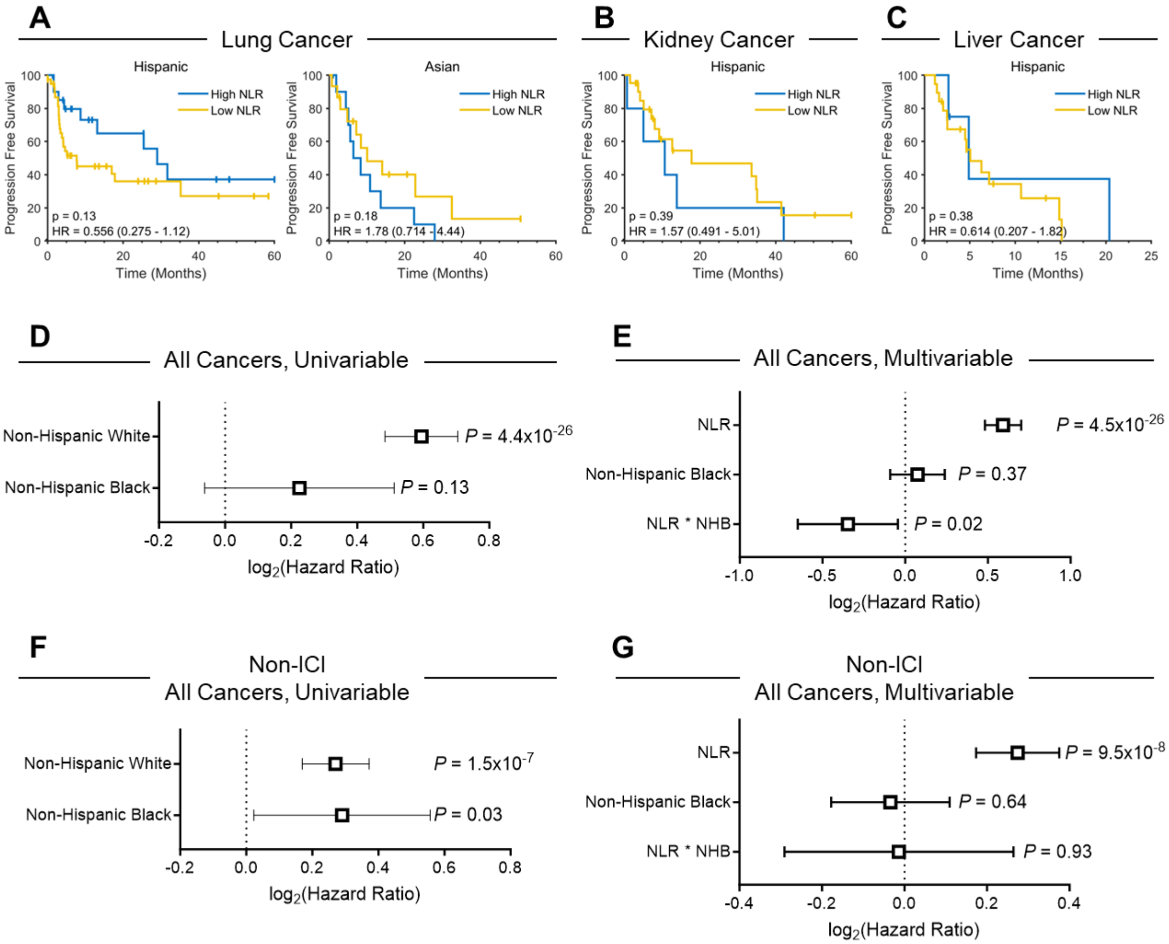

Figure S2. Clinical outcomes based on NLR patients from the Cleveland Clinic cohort.
(A-C) Kaplan-Meier curves following treatment with immune checkpoint inhibition (ICI) stratified by NLR, with high NLR defined as upper tertile for (A) Hispanic (N = 90) and Asian (N = 33) patients with lung cancer, (B) Hispanic patients with kidney cancer (N = 41), and (C) Hispanic patients with liver cancer (N = 25).
(D) Analysis of PFS on ICIs using a univariable Cox proportional hazards model taken as *PFS ~ NLR*, with cancer type as stratification variable, for only non-Hispanic white patients or only non-Hispanic black patients. Non-Hispanic white N = 4081. Non-Hispanic black N = 720.
(E) Analysis of PFS on ICIs using a Cox proportional hazards model taken as *PFS ~ (NLR) + (non-Hispanic black) + (NLR * non-Hispanic black)*, with cancer type as stratification variable. Non-Hispanic white N = 4081. Non-Hispanic black N = 720.
(F) Analysis of PFS on non-ICI treatments using a univariable Cox proportional hazards model taken as *PFS ~ NLR*, with cancer type as stratification variable, for only non-Hispanic white patients or only non-Hispanic black patients. Non-Hispanic white N = 3447. Non-Hispanic black N = 634.
(G) Analysis of PFS on non-ICI treatments using a Cox proportional hazards model taken as *PFS ~ (NLR) + (non-Hispanic black) + (NLR * non-Hispanic black)*, with cancer type as stratification variable. Non-Hispanic white N = 3557. Non-Hispanic black N = 634.

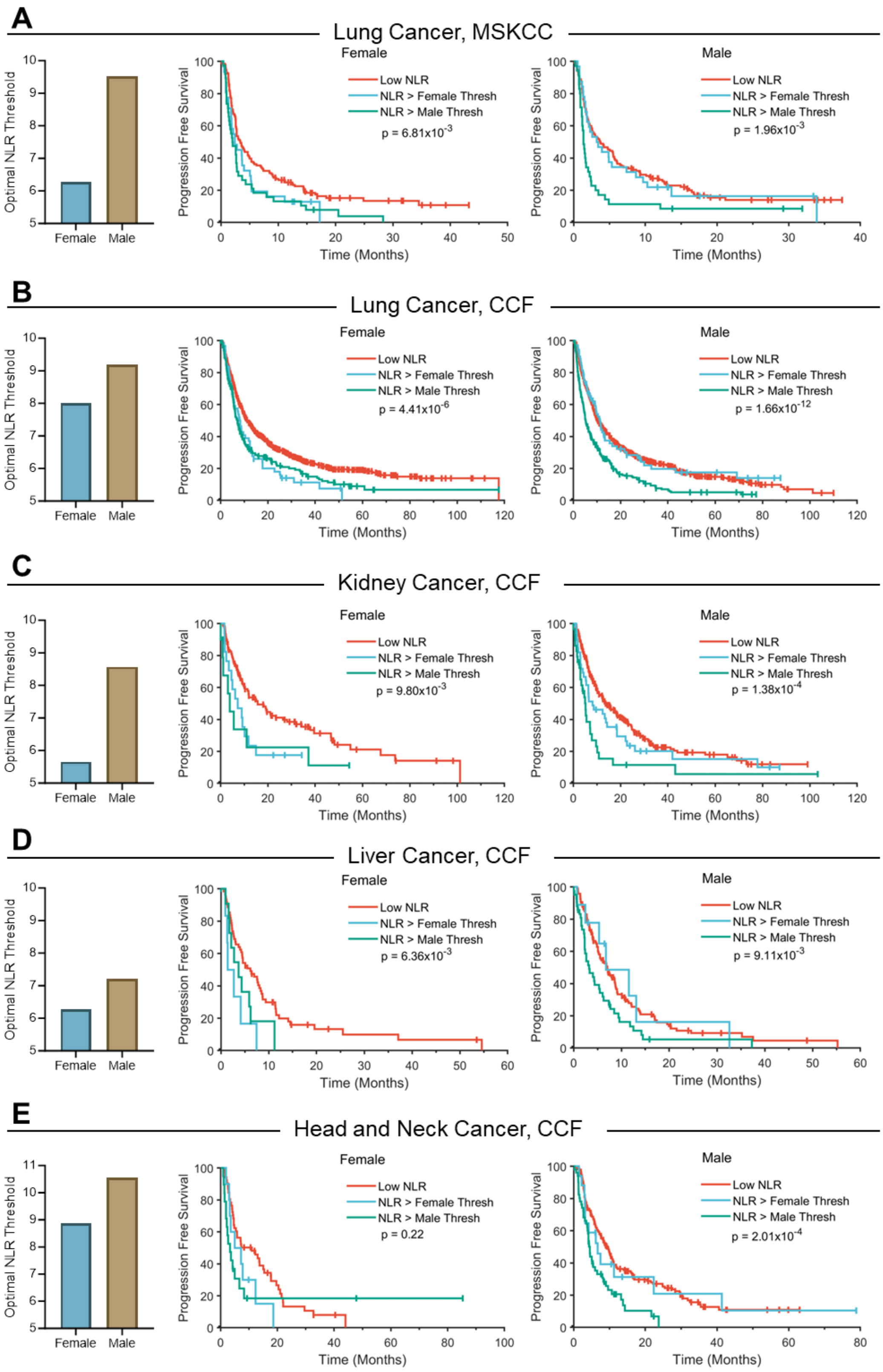

Figure S3. Optimal NLR threshold in non-Hispanic white patients varies by biological sex. Kaplan-Meier curves following treatment with immune checkpoint inhibition (ICI) for female and male non-Hispanic White patients with cancer divided into low NLR, NLR>optimal female

threshold but less than optimal male threshold (NLR > Female Thresh) and NLR>optimal male threshold (NLR > Male Thresh). Optimal NLR threshold was independently determined for each cancer cohort.
(A) Patients with lung cancer from MSKCC. Female N = 214, Male N = 204.
(B) Patients with lung cancer patients from CCF. Female N = 1889, Male N = 1940.
(C) Patients with kidney cancer from CCF. Female N = 254, Male N = 639.
(D) Patients with liver cancer from CCF. Female N = 114, Male N = 234.
(E) Patients with head and neck cancer from CCF. Female N = 93, Male N = 315.

Table S1. NHANES cohort demographics including only complete samples used for analysis.

**Characteristics**	**N = 8,095**
**Sex**	
**Male**	3928 (48.5%)
**Female**	4167 (51.5 %)
**Age: mean (standard deviation)**	49.4 (18.3)
**BMI: median (IQR)**	28.7 (9.0)
**Race/ethnicity**	
**Non-Hispanic White**	2850 (35.2%)
**Non-Hispanic Black**	2034 (25.1%)
**Hispanic**	1844 (22.8%)
**Non-Hispanic Asian**	970 (12.0%)
**Other Race - Including Multi-Racial**	397 (4.9%)

DISCUSSION

In this study, we demonstrate that NLR varies based on sex and race/ethnicity, a relationship which holds after controlling for baseline inflammation levels. We further go on to demonstrate that the predictive utility of NLR may be restricted to NHW patients with cancer, and even within NHW patients the threshold value to identify high NLR may be dependent on patient sex.

Critical outstanding questions include both why NLR is associated with ICI outcomes in NHW patients with cancer, and why is this phenotype diminished in other race/ethnicity groups. We have recently shown that the NLR in circulation is reflected within the tumor microenvironment (1). Lymphocytes, including both B cells and T cells, are required for anti-tumor immunity and ICI activity (2, 3). Tumor-associated neutrophils are key cells in cancer-associated chronic inflammation, having a role in tumorigenesis (4), angiogenesis (5), tumor progression (6), and metastasis (7). Critically for ICI, tumor-associated neutrophils can also directly suppress tumor infiltrating T cells (8, 9). Thus, elevation of intratumoral NLR from a high circulation NLR could likely suppress ICI outcomes. Beyond the intratumoral milieu, in circulation neutrophils have also been shown to support viability of circulating tumor cells to promote cancer dissemination and worsen prognosis (10). If these direct functional roles are the primary drivers, it could imply that either the association between circulation and intratumoral immune landscapes varies by race/ethnicity, or that the functional consequences of altered levels of intratumoral neutrophils/lymphocytes varies by race/ethnicity. To these points, there is existing evidence that neutrophils may function differently based on genetic ancestry. For instance, the Duffy-null associated neutrophil count phenotype (11), previously termed "benign ethnic neutropenia", describes individuals of predominately African descent who have "low" (<1500-2000 cells/µL) neutrophils but are otherwise asymptomatic with no elevated infection risk (12, 13). Current data suggests that this phenotype is driven by a combination of elevated neutrophil margination into tissues and increased effector molecule expression that may result in superior innate immunity against microbial infection (14). The Duffy-null associated neutrophil count and other related phenotypes offer one plausible biological explanation for loss of prognostic relevance of NLR in the context of ICI treatment.

Although this study is retrospective in nature, it is strengthened by validation in datasets across multiple independent cancer centers and multiple cancer types. This study also relied primarily upon binarization at upper tertile to identify NLR high. When attempting to identify optimal sex-specific thresholds, we found variations by cancer type and between cancer centers. Establishing a well-defined NLR threshold value is of critical importance in both ICI therapy and other diseases, but no value has been agreed upon within the field (15). NLR may also be assay dependent. For instance, DNA methylation cytometry cutoffs may vary from classical blood analysis approaches. Notably, though not an analyzed endpoint post-hoc analysis of a prior DNA methylation cytometry study also identifies a decreased NLR in non-Hispanic black individuals (16). The feasibility of establishing a universal value is unclear, but our study provides additional demographic considerations that should be made when exploring any such universal NLR threshold. Beyond demographic considerations, it is also unclear if the NLR threshold should vary by cancer type, or more broadly by disease type. Moreover, race/ethnicity, as used here, is not necessarily reflective of any genetic or biological meaning. To enhance our understanding of this observation, future studies using more precise genetic analysis such as Single Nucleotide Polymorphism (SNP) profiling to understand the fundamental molecular mechanisms mediating the results observed here are required.

In conclusion, our study demonstrates that NLR varies based on patient demographics including sex and race/ethnicity. We found the ability of NLR to predict ICI outcomes was largely restricted to NHW individuals, and that within NHW individuals the optimal threshold varied by sex. Together, these observations highlight the importance of considering patient demographics when implementing clinical biomarkers.

MATERIALS AND METHODS

Sex as a Biological Variable

Our study examined male and female patients, and sex-dimorphic effects are reported.

NHANES Dataset

We analyzed publicly available data from 2017-March 2020 (pre-pandemic) cycle of the National Health and Nutrition Examination Survey (NHANES), a continuous cross-sectional program that monitors the health status of the US population. All data were downloaded through the publicly accessible NHANES website and merged to create a unified dataset. Participants younger than 18 years or those with missing outcome or covariate information were excluded, resulting in a final sample size of 8,095 individuals. Cohort characteristics are given in Table S1.

The neutrophil-to-lymphocyte ratio (NLR) was computed as the ratio of absolute neutrophil and lymphocyte counts. NLR was log-transformed prior to analysis for normality. To capture systemic inflammation, we also created a composite index by defined as C-reactive protein minus serum globulin after log-transformation and z-normalization of both values. Age and body max index (BMI) value were also z-scaled. Sex and race/ethnicity were encoded as categorical factors, with female sex and NHW ethnicity as the reference levels. For race/ethnicity, the "Mexican American" and "Other Hispanic" categories were also combined.

Analysis of NHANES Dataset

Statistical analyses were performed in R v.4.5.0. We fit linear regression models with log(NLR) as the outcome in three stages: (i) five separate univariate models assessing each principal predictor individually; (ii) a multivariable model adjusted for sex, race/ethnicity, age, log-scaled BMI, and the composite inflammation score; and (iii) a second multivariable model that omitted BMI to examine BMI-independent effects. Regression coefficients, 95% confidence intervals, and two-sided p-values were extracted with broom v1.0.8. Additional R packages including tidyverse v2.0.0, foreign v 0.8-90, forestplot v3.1.6, and openxlsx v4.2.8 were used throughout for data processing and visualization.

Memorial Sloan Kettering Cancer Center (MSKCC) Cohort

The MSKCC cohort has been described previously (17). In brief, initial inclusion criteria was patients with solid tumors diagnosed from 2015-2018 who received ICI therapy. After manual curation to ensure data completeness and exclusion of patients with multiple cancers, 1,479 individual patients remained. Notably, the final cohort only included patients with a complete blood count within 30 days before treatment initiation. All studies were approved by the MSKCC Institutional Review Board.

Cleveland Clinic Foundation (CCF) Cohort

All patients with solid tumors treated with immune checkpoint inhibitors at Cleveland Clinic from 2015 through May of 2025 were initially considered for analysis. Patients were excluded if (i) they lacked demographic information, (ii) they lacked complete blood counts within 30 days of therapy initiation, (iii) they were diagnosed with multiple cancers while undergoing immune checkpoint inhibition therapy, or (iv) they only had record of a single treatment administration. Cancer types were identified based on ICD10 codes. Analysis was narrowed to 6 cancer types based on patient availability, specifically lung, kidney, liver, head and neck, cervical, and breast. Progression free survival was defined as time to next treatment or death. The non-ICI cohort was patients receiving any systemic therapy regimen that did not include ICIs, with progression free survival still defined as time to next treatment or death. All studies were approved by the Cleveland Clinic Institutional Review Board.

Analysis of NLR Association with Cancer Outcomes.

For all primary analyses, high NLR was defined as upper tertile and significance assessed by log-rank test. Survival was visualized by Kaplan-Meier curves. Additional analyses pooling all cancer types were performed with a stratified Cox proportional hazards model, taking cancer type as a stratification variable. Power calculations for data from non-Hispanic black patient cohorts were performed using hazard ratios determined data from non-Hispanic white patient cohorts.

To evaluate ability of NLR to predict outcomes following immune checkpoint inhibition therapy across a range of alternative thresholds, assessed NLR threshold values ranging from ~20-80th percentile. The mean and standard deviation of $-\log_{10}$(log-rank P-Value) for PFS following ICI

treatment at each NLR threshold by 7-fold cross validation. This analysis was done both with all patients, and with down-sampling all patient groups to be of equal sample size.

To evaluate sex-specific differences in optimal NLR threshold, optimal NLR threshold was identified by $-\log_{10}$(P-Value) x Hazard Ratio for males and females from each cohort independently. Only NHW patients were used for this analysis. For all cohorts, the female threshold identified was lower than the male threshold. As such, Kaplan-Meier curves were constructed dividing patients into low NLR, NLR values greater than the female threshold and less than the male threshold (NLR > Female Thresh) and NLR values greater than the male threshold (NLR > Male Thresh).

Statistics.

Relationship between NLR and demographic features in NHANES data was analyzed by linear regression in R v4.5.0. Normality was assessed by Q-Q plots, and non-normal variables log-transformed. Survival was primarily assessed by log-rank test and visualized by Kaplan-Meier plots for univariable analysis. For univariable or multivariable analysis using pooled cancer types, a stratified Cox proportional hazards model was used taking cancer type as strata. All tests were two-tailed.

Study Approval.

This study was overseen by the Memorial Sloan Kettering Cancer Center Institutional Review Board (New York, New York, USA) and Cleveland Clinic Foundation Institutional Review Board (Cleveland, Ohio, USA). Informed consent was not required for the data contained within this manuscript as it was considered exempt because the study was secondary research of existing data was for public health research purposes with all samples de-identified.

Data Availability.

National Health and Nutrition Examination Survey (NHANES) data are available from the CDC NHANES website (https://www.cdc.gov/nchs/nhanes/index.html). All other raw data are provided with this manuscript.

AUTHOR CONTRIBUTIONS

W.A. and D.J.M. conceived the project and performed data analysis. W.A., T.D.K., and D.J.M. wrote the manuscript. A.K., C.V., J.L.V, V.M., L.G.T.M., and D.J.M. performed data curation. S.P. advised on statistical analysis. A.A.A., L.G.T.M, S.L.S, and N.L.S. provided scientific input. D.J.M. supervised the project. All authors reviewed and approved the final manuscript.

REFERENCES

1. Mitchell KG, et al. Intratumoral neutrophil-to-lymphocyte ratio is mirrored by circulating neutrophil-to-lymphocyte ratio in non-small cell lung cancer. *J Immunother Cancer*. 2025;13(6).

2. Chow A, et al. Clinical implications of T cell exhaustion for cancer immunotherapy. *Nat Rev Clin Oncol*. 2022;19(12):775–790.

3. Laumont CM, Nelson BH. B cells in the tumor microenvironment: Multi-faceted organizers, regulators, and effectors of anti-tumor immunity. *Cancer Cell*. 2023;41(3):466–489.

4. Jamieson T, et al. Inhibition of CXCR2 profoundly suppresses inflammation-driven and spontaneous tumorigenesis. *J Clin Invest*. 2012;122(9):3127–3144.

5. Nozawa H, Chiu C, Hanahan D. Infiltrating neutrophils mediate the initial angiogenic switch in a mouse model of multistage carcinogenesis. *Proc Natl Acad Sci U S A*. 2006;103(33):12493–12498.

6. Pekarek LA, et al. Inhibition of tumor growth by elimination of granulocytes. *J Exp Med*. 1995;181(1):435–440.

7. Welch DR, et al. Tumor-elicited polymorphonuclear cells, in contrast to “normal” circulating polymorphonuclear cells, stimulate invasive and metastatic potentials of rat mammary adenocarcinoma cells. *Proc Natl Acad Sci U S A*. 1989;86(15):5859–5863.

8. Coffelt SB, et al. IL-17-producing γδ T cells and neutrophils conspire to promote breast cancer metastasis. *Nature*. 2015;522(7556):345–348.

9. Zhang H, et al. Annexin A2/TLR2/MYD88 pathway induces arginase 1 expression in tumor-associated neutrophils. *J Clin Invest*. 2022;132(22):e153643.

10. Szczerba BM, et al. Neutrophils escort circulating tumour cells to enable cell cycle progression. *Nature*. 2019;566(7745):553–557.

11. Merz LE, et al. Development of Duffy Null-Specific Absolute Neutrophil Count Reference Ranges. *JAMA*. 2023;329(23):2088–2089.

12. Merz LE, Achebe M. When non-Whiteness becomes a condition. *Blood*. 2021;137(1):13–15.

13. Atallah-Yunes SA, Ready A, Newburger PE. Benign ethnic neutropenia. *Blood Rev*. 2019;37:100586.

14. Duchene J, et al. Atypical chemokine receptor 1 on nucleated erythroid cells regulates hematopoiesis. *Nat Immunol*. 2017;18(7):753–761.

15. Buonacera A, et al. Neutrophil to Lymphocyte Ratio: An Emerging Marker of the Relationships between the Immune System and Diseases. *Int J Mol Sci*. 2022;23(7):3636.

16. Nissen E, et al. Assessment of immune cell profiles among post-menopausal women in the Women's Health Initiative using DNA methylation-based methods. *Clinical Epigenetics*. 2023;15(1):69.

17. Chowell D, et al. Improved prediction of immune checkpoint blockade efficacy across multiple cancer types. *Nat Biotechnol*. 2022;40(4):499–506.
